# Inhalation of Sulphuric Ether

**Published:** 1847-07

**Authors:** 


*Inhalation of Sulphuric Ether.*—As the controversy of claimants, for priority of discovery, appears to more than keep pace with test and reports on the utility of its administration, a looker on is led to believe the desire for according laurels is deemed paramount to the settlement of the question whether there are many, if any laurels to cope for,—unless they are to be made *specific* in mode suggested by a recent Prussian (Berlin) newspaper; that is, by subscription throughout that country, restricted to two groschens per head—in order that all may have an opportunity to reward rising merit—and the sum total be remitted to Dr. Jackson, whose claims in that country, for the present, appear to be in the ascendant!

Of the many claimants for priority, our attention has not been arrested by any which ante-date lectures on Materia Medica, given by the late Prof. *S. Colhoun*, Jef. Med. Col., Philadelphia—whereof notes in our possession taken during the term 1837-8 run thus:

*Ethers.*—Are made by pouring acids on alcohol—and then distilled. Alcohol should be one-fifth higher than water for medicinal purposes.

*Sulphuric.*—In Hysteria, combined with aqua ammonia—pains in the stomach (spasmodic)—Hooping-Cough, *inhale the vapor. Habitual use, if nervous, may return the complaint.* Local cramps Strangulated hernia—Toothache, one drachm externally.

Removes fatty matters in medical preparation, dose ʒ ½.

*Nitric.—Chloric*, is the best anti-spasmodic.

We wish merely to call attention of parties most interested, to these teachings of the late Prof. COLHOUN, trusting there may be found among his pupils some one with more tenacious memory to fill up these meagre notes—or by reference to the manuscript lectures to give farther information as to the extent which he used inhalation and thus aid in rendering "honor to whom honor."

As a whole, we, individually, would be inclined to look upon the whole controversy as a small affair, did we not see the subject in our periodic journals taking, and tenaciously holding place of matters of more interest. With multiplication of gladiatorial claimants, it is to be hoped this war will be ended by mutual onslaught, and the last remaining individual be restrained within modest bounds—through fear of its repetition from unknown quarters—and, from the plenitude of dear bought wisdom, be induced to concede that at this present, as of old, "there is nothing new under the sun," at least, worth quarrelling about! W. T.

